# Antifungal and cytotoxicity activities of the fresh xylem sap of *Hymenaea courbaril* L. and its major constituent fisetin

**DOI:** 10.1186/1472-6882-14-245

**Published:** 2014-07-16

**Authors:** Maysa Paula da Costa, Marize Campos Valadares Bozinis, Wanessa Machado Andrade, Carolina Rodrigues Costa, Alessandro Lopes da Silva, Cecília Maria Alves de Oliveira, Lucília Kato, Orionalda de Fátima Lisboa Fernandes, Lúcia Kioko Hasimoto Souza, Maria do Rosário Rodrigues Silva

**Affiliations:** 1 Instituto de Patologia Tropical e Saúde Pública, Universidade Federal de Goiás, Rua 235 - s/n - Setor Universitário, CEP: 74605050 Goiânia, Goiás, Brazil; 2 Instituto de Química, Universidade Federal de Goiás, Campus II - Samambaia, Itatiaia, CEP: 74001-970 Goiânia, GO, Brazil; 3 Faculdade de Farmácia, Universidade Federal de Goiás, Praça Universitária, Esq, c/1a Avenida, Qd 62, Setor Universitário, CEP: 74605-220 Goiania, GO, Brazil

**Keywords:** Antifungal activity, Cytotoxicity, *Hymenaea courbaril*

## Abstract

**Background:**

The great potential of plants as *Hymenaea courbaril* L (jatoba) has not yet been throughly explored scientifically and therefore it is very important to investigate their pharmacological and toxicological activities to establish their real efficacy and safety. This study investigated the cytotoxicity of xylem sap of *Hymenaea courbaril* L and its bioactivity against the fungi *Cryptococcus neoformans* species complex and dermatophytes.

**Methods:**

The fresh xylem sap of *H. courbaril* was filtered resulting in an insoluble brown color precipitate and was identified as fisetin. In the filtrate was identified the mixture of fisetinediol, fustin, 3-O-methyl-2,3-*trans*-fustin and taxifolin, which were evaluated by broth microdilution antifungal susceptibility testing against *C. neoformans* species complex and dermatophytes. The fresh xylem sap and fisetin were screened for cytotoxicity against the 3T3-A31 cells of Balb/c using neutral red uptake (NRU) assay.

**Results:**

The fresh xylem sap and the fisetin showed higher *in vitro* activity than the filtrate. The xylem sap of *H. courbaril* inhibited the growth of dermatophytes and of *C. neoformans* with minimal inhibition concentration (MIC) < 256 μg/mL, while the fisetin showed MIC < 128 μg/mL for these fungi. Fisetin showed lower toxicity (IC_50_ = 158 μg/mL) than the fresh xylem sap (IC_50_ = 109 μg/mL).

**Conclusion:**

Naturally occurring fisetin can provide excellent starting points for clinical application and can certainly represent a therapeutic potential against fungal infections, because it showed *in vitro* antifungal activity and low toxicity on animal cells.

## Background

Covering about a quarter of Brazil, the cerrado is the nation’s second largest biome after the Amazon and it is characterized by different vegetation physiognomies, comprising savanna-like formations, forest forms, and also gallery forests [1,2]. The endemic plants are adapted to drought and fire, and this may be responsible for the large diversity in their secondary metabolites. In central Brazil, a substantial part of the population relies on medicinal plants for primary health care. The great potential of these plants has not yet been thoroughly explored scientifically and therefore it is very important to investigate the pharmacological and toxicological activity of these herbs to establish their real efficacy and safety. From our screening program, we selected *Hymenaea courbaril* L. (Fabaceae) a medicinal species popularly known in Brazil as ‘jatoba’ which has a long history of use as medicinal plant by indigenous tribes of the Amazon Basin and also in caatinga and cerrado communities. The jatoba bark is used to give energy and stamina, as well as a tonic for the respiratory tract and for the treatment of urinary systems. The fruit is used to treat mouth ulcers, and the leaves and wood are used for diabetes. The “jatoba” is also used popularly for cystitis, hepatitis, prostatitis, coughs, bronchitis, for stomach problems as well as to treat mycoses of nails [3].

Scientific studies on the medicinal properties of *H. courbaril* revealed antimicrobial activity against Gram-positive bacteria and dengue virus type-2 [4,5]. The secondary metabolites of this plant showed the presence of flavonoid fisetin as the major compound [6], probably responsible for the antimicrobial properties. Fisetin is a natural flavonoid of interest in cancer prevention and therapy because this compound is relatively non toxic compared to other chemotherapeutic agents used in cancer therapy [7-9]. Fisetin possesses antioxidant and anti-inflammatory activity and was found to be cytotoxic and antiangiogenic *in vitro*[10-12]*.* After systemic administration in mice, fisetin has shown interesting antitumor activity in several cancer models, including prostate, teratocarcinoma and lung carcinoma [12,13]. According to Touil *et al*. [12] fisetin was found to be several times less cytotoxic towards normal NIH 3T3 cells when compared to tumor cells, and this could give to this compound an important *in vivo* advantage in terms of therapeutic index.

The fungal infections represent a significant problem to health and are one of the causes of morbidity and mortality in the world. The dermatophytosis caused by filamentous fungi such as *Microsporum* spp, *Trichophyton* spp and *Epidermophyton floccosum* represents a serious medical problem affecting about 20-25% of the world’s population [14]. This mycosis affects mainly human scalp, feet and hands, nails and interdigital areas involving the patient’s life quality [15]. The cryptococcosis caused by yeasts of *C. neoformans* species complex is an opportunistic infection that frequently causes meningoencephalitis in patients with impaired immune systems [16-19]. In Brazil, cryptococcosis is diagnosed in course at the time of disease in about 6% of acquired immune deficiency syndrome (AIDS) patients [20,21]. According to the Center for Disease Control Prevention (CDC) cryptococcal meningitis kills about 624,000 people each year [19]. Drugs used for treatment of cryptococcosis and dermatophytosis have considerable side-effects and adverse effects accompanied by the development of resistance by microorganism with reduced ability to clear completely the infection [22-25].

Some reports have described the biological activities of leaves, seed and trunk resin of *H. courbaril*[26-29], but there is a lack of information on the xylem sap. In the present work, we have focused on the phytochemical study, cytotoxicity and antifungal activity of the fresh xylem sap of *H. courbaril* and its major compound fisetin (1).

## Methods

### Extraction and isolation

The fresh xylem sap (extracted from hole through the bark to heartwood from jatoba tree, 250 mL), of *H. courbaril* was purchased in “Vaga-Fogo” Farm, Pirenopolis, Goiás, Brazil. The insoluble brown color precipitate was filtered through filter paper and it was analyzed by ^1^H and ^13^C NMR [Varian Mercury plus BB spectrometer, operating at 300.059 MHz (^1^H) and 75.458 MHz (^13^C) using CDCl_3_ solutions with TMS as an internal standard] and was identified as fisetin (1, 10 mg).

The filtrate of fresh xylem sap was lyophilized, and the dried extract (2.6 g) was fractionated on silica gel 60 using a hexane/ethyl acetate mixture of increasing polarity to yield 30 mg of the mixture of 4 compounds wich were eluted with hexane/ethyl acetate (20:80) and it were identified by the 1D and 2D NMR analysis. Fisetinediol (2); fustin (3); 3-O-methyl-2,3-*trans*-fustin (4) and taxifolin (5) were identified in the mixture**.** The NMR data were compared to literature (Mujwah *et al.*[30], Piacente *et al.*[31] and Baderschneider & Winterhalter [32]).

### Structural elucidation

#### Fisetin (1)

^1^H NMR (300 MHz, MeOD) 6.88 (d; 8.1, H5′); 6.91 (d; 2.1, H8); 6.92 (dd; 8.7; 2.1, H6); 7.66 (dd; 8.1; 2.1, H6′); 7.76 (d; 2.1, H2′); 7.98 (d; 8.7, H5); ^13^C NMR (75 MHz, MeOD): 103.0 (C8); 115.4 (C10); 115.9 (C2′); 116.0 (C5′ and C6); 121.6 (C6′); *124.2 (C1′); *127.6 (C5); 138.6(C3); *147.1 (C2, C3′and 4′); 158.5 (C9); *164.0 (C7); 173.4 (C4).

**Figure 1:**
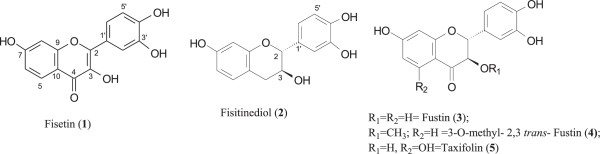
Chemical structures of the compounds 1-5.

The profile in HPLC of crude extract was carried on a Shim-Pack CLC-ODS (H) (4.6 mm × 25 cm) was performed on a Shimadzu LC8A system, using 30% metanol/H_2_O acidified with 0.3% formic acid as eluent. The Figure 1 shows a comparison of fisetin **(1)** and crude extract sap.

#### Fisetinediol (2)

^1^H NMR (300 MHz, MeOD) 2.67 (dd; 15,9; 7,8; H4a); 2.87 (dd; 15.9; 5.1; H4b); 3.99 (ddd; 7.8; 7.2; 5.1; H3); 4.63 (d; 7.2; H2); 6.27 (d; 2.4; H8); 6.33 (dd; 8.1; 2.4, H6); 6.69 (dd, 8.1; 2.1; H6′); 6.75 (d; 8.1; H5′); 6.81 (d; 2.1; H2′); 6.85 (d; 8.1;H5). ^13^C NMR (75 MHz, MeOD): 33.1 (C4); 83.0 (C2); 68.8 (C3); 103.6 (C8); 109.4 (C6); 112.5 (C10); 115.1 (C2′); 116.1 (C5′); 119.8 (C6′); 131.3 (C5); 132.2 (C1′); 146.3 (C3′);146.3 (C4′);156.2 (C9); 157.9 (C7);

#### Fustin (3)

^1^H NMR (300 MHz, MeOD) 4.47 (d; 11.7, H3); 4.92^#^ (H2); 6.26 (d; 2.1, H8); 6.35 (dd; 8.9; 2.1, H6); 6.83 (m, H6′); 6.89 (d; 8.4, H5′); 6.97 (d; 2.1, H2′); 7.67 (d; 8.9, H5); ^13^C NMR (75 MHz, MeOD): 74.6 (C3); 85.5 (C2); 103.8 (C8); 109.3 (C6); *114.0 (C10); 115.9 (C2′);116.2 (C5′);120.9 (C6′);*129.9 (C1′);133.5(C5); *146.2 (C3′);*147.1 (C4′);*166.9 (C9);*167.0 (C7); *205.0 (C4).

#### 3-O-methyl-2,3-*trans*-fustin (4)

^1^H NMR (300 MHz, MeOD) 3.30^#^ (OCH_3_); 4.16 (d; 10.2, H3); 5.06 (d; 10.2, H2); 6.32 (d; 2.1, H8); 6.51 (dd; 8.7; 2.1, H6); 6.79 (d; 8.1, H5’); 6.83 (m, H6’); 6.93 (d; 2.1, H2’); 7.69 (d; 8.7, H5); ^13^C NMR (75 MHz, MeOD): 60.6 (OCH_3_); 83.4 (C3); 84.2 (C2); 103.7 (C8); 112.2 (C6); *113.8 (C10);115.6 (C2′);116.0 (C5′); 120.4 (C6′);*129.9 (C1′); 130.1 (C5); *146.3 (C3′);*147.1 (C4′);*166.9 (C9); *167.0 (C7); *205.0 (C4).

#### Taxifolin (5)

^1^H NMR (300 MHz, MeOD) 4.49 (d; 11.4, H3); 4.89^#^ (H2); 5.87 (d; 2.1; H8); 5.91 (d; 2.1; H6); 6.79 (d; 8.1, H5′); 6.83 (m, H6′); 6.95 (d; 2.1, H2′). ^13^C NMR (75 MHz, MeOD): 73.7 (C3);85.1 (C2); 97.4 (C6); 96.4 (C8); 101.9 (C10); 115.8 (C2′);116.0 (C5′);120.9 (C6′);129.9 (C1′);147.1 (C3′ and C4′); 164.0 (C9); *166.8 (C5); *169.0 (C7).

*The signal could be exchanged. ^#^These signals were under the solvent signal, then were attributed by HSQC experiment.

### Fungal strains

The microorganisms used in this study were obtained from the fungal collection of the Laboratory of Mycology (IPTSP - UFG), from previously work performed in Goiânia-GO at the ‘Hospital das Clínicas’ (HC-UFG) and the ‘Hospital de Doenças Tropicais’ approved by the respective hospitals ethics committees with protocols numbers 007/2004 and 065/2008. These microorganisms comprised 18 strains of dermatophytes and 26 of *C. neoformans* species complex. The fungi were maintained on Sabouraud dextrose agar at -70°C (Difco) and subcultured on the same medium for 72 hours before testing.

### *In vitro* susceptibility testing

The *in vitro* activity of the sap of *H. courbaril* and the isolated compounds and mixture was evaluated using the broth microdilution method, as described in Clinical and Laboratory Standards Institute (CLSI) documents M27-A3 for yeasts and M38-A2 (with some modifications) for dermatophytes [33-35].

In a previous experiment, xylem sap and the compounds **1-5** were screened broth microdilution method against six isolates of dermatophytes and six of yeasts of *C. neoformans* species complex, with concentrations ranging from 256 to 0.25 μg/mL for both fungi. Posteriorly, according to results obtained, *in vitro* susceptibility tests were also performed using fresh xylem sap and fisetin against 18 dermatophytes and 26 yeasts, with concentrations ranging from 256 to 0.25 μg/mL for fresh xylem sap and from 128 to 0.125 μg/mL for fisetin.

Cell suspensions of *C. neoformans* were prepared from 3-day-old cultures in Sabouraud’s dextrose agar at 28°C in sterile saline (0.85%), and the optical density was adjusted using a spectrophotometer to 85% transmittance at a wavelength of 530 nm. This suspension was diluted to 1:50 and then 1:20 in RPMI 1640 medium (Sigma Chemical Co., St. Louis, MO, USA) buffered to a pH of 7.0 with 0.165 mol/L MOPS (Sigma Chemical Co.) to obtain a final inoculum of approximately 1 to 5×10^3^ CFU⁄mL [33].

The inocula of dermatophytes were performed according to Santos *et al.*[35]. Briefly, the isolates were subcultured in potato dextrose agar at 28°C for 7 days to produce conidia. The fungal colonies were then covered with 5 mL of sterile saline (0.85%), and suspensions were made by scraping the surface with the tip of a Pasteur pipette. The resulting conidia and hyphal particles were transferred to a sterile tube and allowed to settle for 15–20 min at room temperature. The density of the suspension was adjusted using a spectrophotometer at wavelength of 520 nm to a transmittance of 70 to 72%. The resulting suspension was diluted to 1:50 in RPMI medium to obtain the final inoculum of approximately 2 to 4×10^4^ CFU⁄mL [34].

The MIC values were determined after 5 days of incubation at 28°C for dermatophytes and after 72 h at 37°C for yeasts. The MIC was defined as the lowest concentration showing 100% growth inhibition compared with growth in the control. *Candida parapsilosis* ATCC 22019 and *C. neoformans* ATCC 28957 were used as controls. Itraconazole (Sigma Chemical Co.) and DMSO diluted in the same way were included as quality controls. Each experiment was performed in duplicate.

### *In vitro* Cytotoxicity

Cell viability was evaluated by neutral red uptake (NRU) according to Borenfreund and Puerner [36], modified by NICEATM-ICCVAM [37]. Briefly, a Balb/c 3T3-A31 fibroblast cell line was grown in DMEM-1640 [(Sigma™, St Louis, MO), high glucose medium containing 10% FBS (Cultilab™)]. The cells were harvested with trypsin/EDTA and seeded (100 μL/well) at an initial density of 3×10^4^ cells/mL into a 96-well plate and incubated overnight. After 24 hours of incubation at 37°C, the cells were treated with eight different concentrations (256 to 2 μg/mL) of fresh xylem sap or fisetin diluted in DMEM medium and incubated for 48 h. The medium was aspirated and replaced with 250 μL per well (including blank) of neutral red (NR) solution. After 3 h incubation (37 ± 1^º^C, 90 ± 10% humidity, 5 ± 1% CO_2_/air) the NR medium was removed, and the cells were washed with pre-warmed PBS. The PBS was decanted and 100 μL of an aqueous solution of 1% acetic acid: 50% ethanol (v/v) was added to each well to extract the dye. After rapid shaking (20 min) in a microtitre plate shaker, the absorbance was read at wavelength of 540 nm.

Cytotoxicity tests were performed at least three times, using six wells for each concentration of fresh xylem sap or of fisetin. The data for the dose-response cytotoxicity curves are presented as the arithmetic mean and standard deviation. Linear regression analysis was used to compute the concentration that reduced absorbance by 50% (IC_50_). The NRU assay results are expressed as the percentage uptake of neutral red dye by lysosomes.

## Results

The screening of *in vitro* antifungal susceptibility of fresh xylem sap, fisetin (**1)** and the mixture of fisinetidiol (**2**), fustin (**3**), 3-O methyl, 2,3-*trans*-fustin (**4**) and taxifolin (**5**) (Figure 1) of *H. courbaryl* showed that both yeasts (6 strains) and dermatophytes (6 strains) were more susceptible to fresh xylem sap and to fisetin than the mixture of compounds. In this screening, all the tested strains showed susceptibility to fisetin at concentrations of 32-128 μg/mL, while for xylem sap this concentration ranged of 32-256 μg/mL.

In posterior evaluation against 44 isolates (18 dermatophytes and 26 yeasts), the fresh xylem sap of *H. courbaril* inhibited the growth of dermatophytes and of *C. neoformans* species complex with MIC values of 8-256 μg/mL and geometric means of 64-181 μg/mL, while the fisetin showed a MIC of 4-128 μg/mL and geometric means of 21.5-128 μg/mL for the fungi tested (Table 1). Quality controls performed with itraconazole showed MIC of 1 μg/mL and of 0.25 μg/mL for all isolates of dermatophytes and *C. neoformans* species complex, respectively.

**Table 1:** ***In vitro *** **antifungal activity of the sap and fisetin of *H. courbaril***

**Isolates**	**Minimum inhibitory concentration (μg/mL)**							
	**Fresh xylem sap**				**Fisetin**			
	**Range**	**MIC**_**50**_	**MIC**_**90**_	**GM**	**Range**	**MIC**_**50**_	**MIC**_**90**_	**GM**
Dermatophytes (n)								
*M. gypseum* (2)	64-128	128	128	90.5	64-128	128	128	90.5
*T. mentagrophytes* (8)	32-128	64	128	83	32-128	64	128	69.8
*T. rubrum* (7)	32-128	64	128	64	4-64	32	64	21.5
*T. tonsurans* (1)	128	128	128	128	128	128	128	128
Yeasts (n)								
*C. gattii* (4)	128-256	128	256	181	128	128	128	128
*C. neoformans* (22)	8-256	64	256	68,2	8-128	64	128	48.2

MIC_50_ - inhibit the growth of 50% of isolates;

MIC_90_ - Minimal inhibitory concentration to inhibit the growth of 90% of isolates;

GM = Geometric Mean.

**Figure 2:**
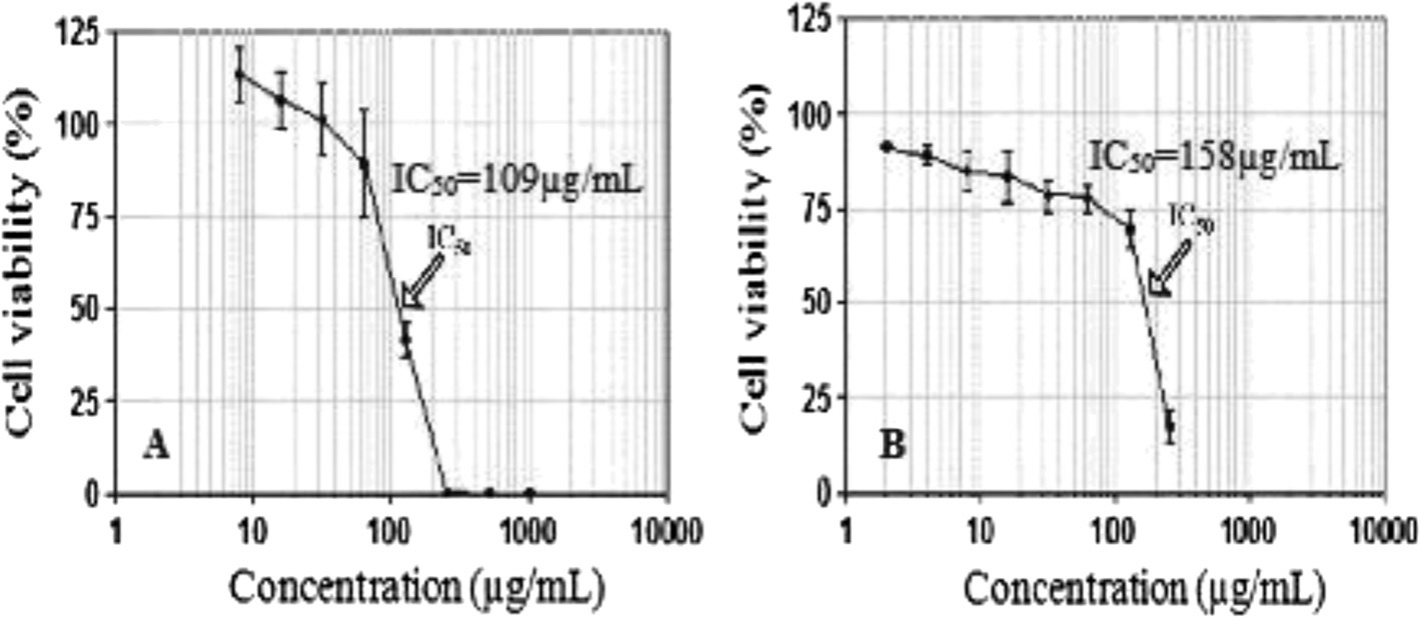
**Cell viability of 3T3-A31 fibroblasts exposed to different concentrations of fresh xylem sap of *H. courbaril *** **(A) and of fisetin (B).**

**Figure 3:**
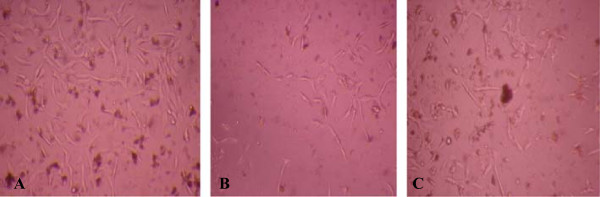
**The cells of Balb/C untreated and treated with xylem sap and fisetin. A**- The cell-fibroblast Balb/c 3T3-A31 untreated. **B**- Cells treated with fresh xylem sap of *H. courbaril* at a concentration of 64 μg/mL. **C**- Cells treated with fisetin **1** at a concentration of 128 μg/mL. Photomicrograph at 200X by inverted microscope.

The results of *in vitro* cytotoxicity showed lower toxicity of fisetin than of fresh xylem sap against 3T3-A31 cells of Balb/c with an IC_50_ of 109 μg/mL for fresh xylem sap of *H. courbaril* and of 158 μg/mL for fisetin. The percentage of growth inhibition of the 3T3-A31 cells increased with increasing concentrations of fresh xylem sap or fisetin as shown in Figure 2. Reduction in the amount of fibroblast cells 3T3-A31 treated with the fresh xylem sap and fisetin visualized by inverted light microscope is showed in Figure 3.

## Discussion

Antifungal drugs available do not completely satisfy the medical necessity due to problems such as spectrum, potency, security, and their pharmacokinetic properties. Nowadays, there is an increased interest in searching for new antifungal compounds that function as selective and low toxic**.** Natural products may be used as templates for development of new drugs by the pharmaceutical industry and compounds extracted of plants have been considered the major resources of bioactive agents. It was estimated that at least 12000 active compounds have been isolated from medicinal plants as antimicrobial agents representing less than 10% of the total [38,39].

The present work has demonstrated the antifungal activity of fisetin and fresh xylem sap from *H. courbaril*, species largely found in Brazil, against yeasts of *C. neoformans* species complex and filamentous fungi as dermatophytes. Numerous assay systems and organisms have been used to screening plant extracts and constituents of active plants for antimicrobial activity. The broth microdilution method used in this work has several advantages. This method is quantitative, allows the use of small quantities of compounds or plant extracts as well as culture media [40]. It was observed MIC values below 256 μg/mL for fisetin and for fresh xylem sap against these fungi. There is no consensus in the literature on the MIC values of a plant extract which qualifies it as promising for fractionation. According to Kuete [40], the antimicrobial activity of extracts can be classified as follows: significant if MIC values are below100 μg/ml, moderate when 100 < MIC < 625 μg/ml and weak if MIC > 625 μg/ml. Therefore, the overall antifungal activity exhibited in this study varied from moderate to significant. Previous studies have shown that flavonoids-rich extracts possesses antimicrobial activity [41,42]. Although the identification of mechanism action of flavonoids has been discussed in the literature and there are indications that antimicrobial proprieties of flavonoids are due to its interference with the specific intracellular of enzymes surface [43-45], a new studies to the identification of its mechanism action is still necessary.

In the present study, it was used NRU assay to determine the cytotoxic effect of fisetin and fresh xylem sap of *H. courbaril* in Balb/c 3T3-A31 fibroblast cell line to determine their IC_50_. Cell viability evaluation of fisetin showed IC_50_ of 158 μg/mL and of sap of 109 μg/mL, with low reduction of number of cells visualized by inverted microscope (Figure 3). In this way, fisetin and xylem sap had good results of IC_50_ when compared to their MIC against *Cryptococcus* and dermatophytes. The xylem sap of *H. courbaril* inhibited the growth of dermatophytes and of the yeast *C. neoformans* species complex with MIC below 256 μg/mL, while the fisetin showed MIC below 128 μg/mL. Cell cytotoxicity assays are commonly used *in vitro* bioassay methods to predict the toxicity of substances in various tissues, because they demonstrate the degree of damage caused by the chemical [46].

The results obtained in this work, showed lower toxic effect of fisetin to mouse fibroblast cell line and higher activity against fungi than fresh xylem sap of *H. courbaril*. The naturally occurring flavonol, fisetin (C_15_H_10_O_6_), is produced ubiquitously in the plant kingdom and may be found in high concentrations in certain food plants, most notably grape, onion and cucumber [47].

## Conclusion

Thus, fisetin, has advantage over the antifungals used commercially and can certainly represent a therapeutic potential due to *in vitro* antifungal activity and low toxicity on animal cells.

Although the results have suggested that fisetin may be useful as antifungal drug, further studies of pharmacokinetics and pharmacodynamics aspects are needed for utilization of this natural product.

## Competing interest

The authors have no conflict of interests to declare.

## Authors’ contributions

MPC and MRRS: conceived and designed the work, drafted the manuscript, performed the experiments and contributed in analysis of data. MCVB and WMA: Contributed in the analysis of cytotoxicity. CMAO and LK provided the plant material and carried out the compounds extraction and HPLC analysis. CRC, LKHS and OFLF contributed in the analysis and interpretation of experiment data and participated in manuscript preparation. All authors gave their approval for the final version of the manuscript to be published.

## Pre-publication history

The pre-publication history for this paper can be accessed here:

http://www.biomedcentral.com/1472-6882/14/245/prepub
